# Supplementary material for: High Diagnostic Accuracy of a Novel Lateral Flow Assay for the Point-of-Care Detection of SARS-CoV-2
Source: Biomedicines. 2022 Jun 30;10(7):1558. doi: 10.3390/biomedicines10071558 (PMC9312898; doi:10.3390/biomedicines10071558)
Supplement: Supplementary file 1 [file biomedicines-10-01558-s001.zip › biomedicines-1792237-supplementary.pdf]

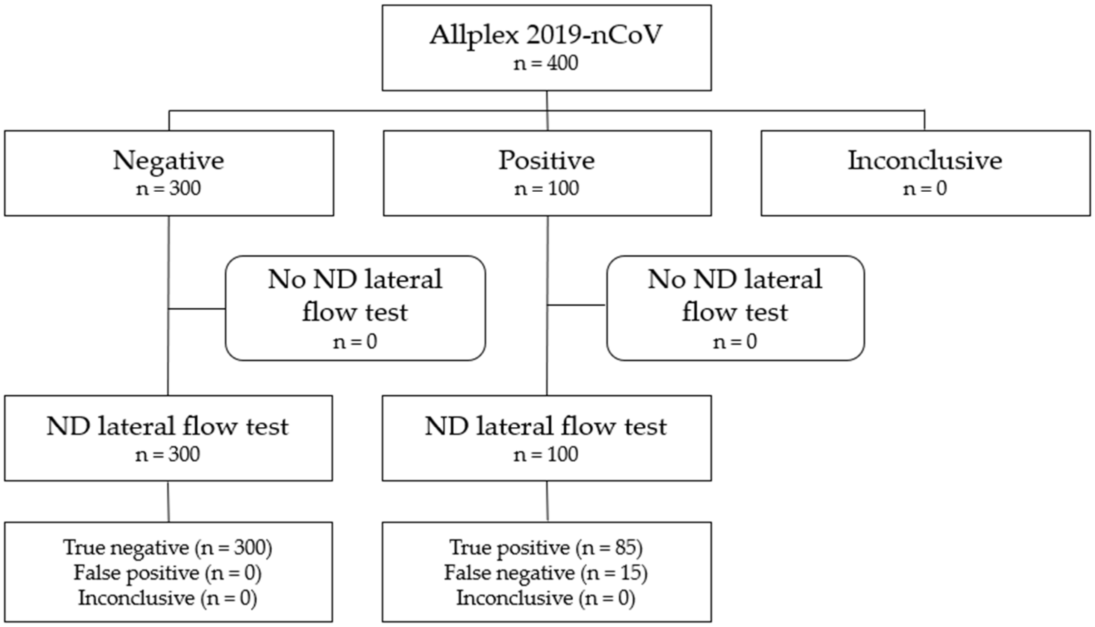

Figure S1. STARD (Standards for Reporting of Diagnostic Accuracy Studies) flowchart.

Table S1. STARD (Standards for Reporting of Diagnostic Accuracy Studies) checklist.

Section & Topic	No	Item	Reported in section (§)
**TITLE OR ABSTRACT**			
	**1**	Identification as a study of diagnostic accuracy using at least one measure of accuracy (such as sensitivity, specificity, predictive values, or AUC)	Title (NA)
**ABSTRACT**			
	**2**	Structured summary of study design, methods, results, and conclusions (for specific guidance, see STARD for Abstracts)	Abstract (NA)
**INTRODUCTION**			
	**3**	Scientific and clinical background, including the intended use and clinical role of the index test	1 (1–6)
	**4**	Study objectives and hypotheses	1 (6)
**METHODS**			
Study design	**5**	Whether data collection was planned before the index test and reference standard were performed (prospective study) or after (retrospective study)	2.2 (2)
Participants	**6**	Eligibility criteria	2.2 (2)
	**7**	On what basis potentially eligible participants were identified (such as symptoms, results from previous tests, inclusion in registry)	2.2 (2)
	**8**	Where and when potentially eligible participants were identified (setting, location and dates)	2.2 (1)
	**9**	Whether participants formed a consecutive, random or convenience series	2.2 (2)
Test methods	**10a**	Index test, in sufficient detail to allow replication	2.3 (1–2)
	**10b**	Reference standard, in sufficient detail to allow replication	2.4 (1–2)
	**11**	Rationale for choosing the reference standard (if alternatives exist)	2.4 (1)
	**12a**	Definition of and rationale for test positivity cut-offs or result categories of the index test, distinguishing pre-specified from exploratory	2.3 (2)
	**12b**	Definition of and rationale for test positivity cut-offs or result categories of the reference standard, distinguishing pre-specified from exploratory	2.4 (1)
	**13a**	Whether clinical information and reference standard results were available to the performers/readers of the index test	2.2 (2)
	**13b**	Whether clinical information and index test results were available to the assessors of the reference standard	2.2 (2)
Analysis	**14**	Methods for estimating or comparing measures of diagnostic accuracy	2.5 (1)
	**15**	How indeterminate index test or reference standard results were handled	2.2 (2) and 3 (1)
	**16**	How missing data on the index test and reference standard were handled	2.2 (2)
	**17**	Any analyses of variability in diagnostic accuracy, distinguishing pre-specified from exploratory	2.4 (1) and 2.5 (1)

	**18**	Intended sample size and how it was determined	2.2 (2)
**RESULTS**			
Participants	**19**	Flow of participants, using a diagram	Figure S1
	**20**	Baseline demographic and clinical characteristics of participants	3 (1)
	**21a**	Distribution of severity of disease in those with the target condition	NA
	**21b**	Distribution of alternative diagnoses in those without the target condition	NA
	**22**	Time interval and any clinical interventions between index test and reference standard	2.2 (2)
Test results	**23**	Cross tabulation of the index test results (or their distribution) by the results of the reference standard	3 (2) and Figures 1 and S1
	**24**	Estimates of diagnostic accuracy and their precision (such as 95% confidence intervals)	3 (Tables 1–2)
	**25**	Any adverse events from performing the index test or the reference standard	NA
**DISCUSSION**			
	**26**	Study limitations, including sources of potential bias, statistical uncertainty, and generalisability	4 (4)
	**27**	Implications for practice, including the intended use and clinical role of the index test	4 (1–3)
**OTHER INFORMATION**			
	**28**	Registration number and name of registry	NA
	**29**	Where the full study protocol can be accessed	NA
	**30**	Sources of funding and other support; role of funders	Funding (1)
